# Light color efficiency-balanced trans-palpebral illumination for widefield fundus photography of the retina and choroid

**DOI:** 10.1038/s41598-022-18061-7

**Published:** 2022-08-16

**Authors:** Taeyoon Son, Jiechao Ma, Devrim Toslak, Alfa Rossi, Hoonsup Kim, R. V. Paul Chan, Xincheng Yao

**Affiliations:** 1 Department of Bioengineering, University of Illinois at Chicago, Chicago, IL 60607 USA; 2 Department of Ophthalmology, Antalya Training and Research Hospital, Antalya, Turkey; 3 Department of Ophthalmology and Visual Sciences, University of Illinois at Chicago, Chicago, IL 60612 USA

**Keywords:** Biomedical engineering, Translational research

## Abstract

A wide-field fundus camera, which can selectively evaluate the retina and choroid, is desirable for better detection and treatment evaluation of eye diseases. Trans-palpebral illumination has been demonstrated for wide-field fundus photography, but its application for true-color retinal imaging is challenging due to the light efficiency delivered through the eyelid and sclera is highly wavelength dependent. This study is to test the feasibility of true-color retinal imaging using efficiency-balanced visible light illumination, and to validate multiple spectral imaging (MSI) of the retina and choroid. 530 nm, 625 nm, 780 nm and 970 nm light emission diodes (LED)s are used to quantitatively evaluate the spectral efficiency of the trans-palpebral illumination. In comparison with 530 nm illumination, the 625 nm, 780 nm and 970 nm light efficiencies are 30.25, 523.05, and 1238.35 times higher. The light efficiency-balanced 530 nm and 625 nm illumination control can be used to produce true-color retinal image with contrast enhancement. The 780 nm light image enhances the visibility of choroidal vasculature, and the 970 nm image is predominated by large veins in the choroid. Without the need of pharmacological pupillary dilation, a 140° eye-angle field of view (FOV) is demonstrated in a snapshot fundus image. In coordination with a fixation target, the FOV can be readily expanded over the equator of the eye to visualize vortex ampullas.

## Introduction

Fundus photography is indispensable for screening, diagnosis, and management of eye diseases in ophthalmology. Because many eye diseases can affect both central and peripheral regions of the retina, a wide-field fundus photography has demonstrated its utility in the clinical management of eye diseases such as diabetic retinopathy (DR)^1^, age-related macular degeneration (AMD)^2^, glaucoma^3^, hypertensive retinopathy^4^, retinal detachments^5^, and vascular pathologies (vascular occlusions, vasculitis, etc.)^6^ with ocular metastasis. In addition to retinal imaging, choroidal imaging can provide a valuable supplement to traditional retinal imaging for better management of choroidal disorders. For example, AMD may produce choroidal neovascularization (CNV)^7^. Diabetic choroidopathy (DC) may induce loss of choriocapillaris (CC), tortuous blood vessels and reduction of blood flow in sub foveal choroidal vasculature^8^. Also, significant choroidal vascularity index (CVI) reduction has been reported in glaucoma and retinitis pigmentosa^9^. Multispectral imaging (MSI) technology, which employs multiple wavelengths from visible to near infrared, has been reported to visualize choroidal fundus. However, currently available MSI systems have limited FOV, typically 45° visual-angle (68° eye-angle)^10–12^.

It is technically difficult to construct wide-field fundus cameras, due to its illumination mechanisms^13^. Conventional fundus cameras utilized trans-pupillary illumination; a donut-shape patterned illumination delivered to the interior of the eye^14^. Based on the Gullstrand-Principle, the illumination and imaging path must be separated^15^. Otherwise, the illumination beam cause severe reflection at the cornea and crystalline lens consequently degrading image quality. Therefore, trans-pupillary illumination limits the field of view (FOV), typically 30° or 45° visual-angle (45°–68° eye-angle), of fundus images because only a small portion of the pupil is used for imaging purposes and the peripheral area of the pupil must be used for illumination^16^. For wide-field fundus imaging, pupillary dilation is typically required. Pharmacological pupillary dilation cause patients to experience light glare and focusing difficulty for hours and even days in some cases. The miniaturized indirect ophthalmoscopy has been developed for wide-field fundus imaging by minimizing the illumination portion of the available pupil^17,18^. A 67° visual-angle (101° eye-angle) FOV nonmydriatic fundus imaging has been achieved by utilizing NIR guidance for imaging alignment and focus adjustment. Daytona and California series (Optos, Dunfermline, UK), scanning laser ophthalmoscope (SLO) based fundus imager, has been established for ultra-wide field fundus imaging with a 134° visual-angle (200° eye-angle) FOV^19,20^. However, it involves multiple laser light sources and a complicated scanning system which increase the complexity and device cost. Also, the eyelashes and eyelids may obstruct the peripheral area of these fundus images. The visual-angle has been used to present the FOV of conventional fundus photography. Recently, the eye-angle emerges as the unit in the wide field fundus photography which creates confusion about FOV interpretation. There is an effort to understanding the relationship between visual-angle and eye-angle^21^. In this study, we provide both visual-angle and eye-angle to avoid confusion.

Trans-pars-planar illumination has been investigated to expand FOV of fundus images, without pharmacological pupillary dilation^14,16,22^. The pars-plana is a posterior part of the ciliary body which lacks muscle, blood vessels and pigmentation. Therefore, it can be used as a window to deliver the illumination light in the eye. Both contact and contact free trans-pars-planar illumination has been demonstrated. Wang et al. archived 60° visual-angle (90° eye-angle) wide-field fundus images through contact free trans-pars-planar illumination. It was also demonstrated that the brightness analysis of fundus images collected from different locations to confirms the transparency of the pars-plana^16^. Toslak et al. validated the contact mode trans-pars-planar illumination in a portable ultra-wide field (134° visual-angle; 200° eye-angle) fundus camera for pediatric and adult subjects^14,22^. By freeing the entire pupil for imaging purposes only and having the lens contact the eye, the fundus image enabled visualization of both the central and peripheral retina up to the ora serrata. However, the scleral contact of the trans-pars-planar illuminator may create clinical complications, such as possible contact inflammation and then sterilization requirement.

Trans-palpebral illumination has been demonstrated as one scleral contact-free alternative to the trans-pars-planar illumination for wide-field fundus photography^23^. Instead of direct contact of the trans-pars-planar illuminator to the sclera, the trans-palpebral illuminator delivers the light through the eyelid, promising a simple solution to achieve affordable wide-field imaging without consideration of contamination by contact of the lens directly on the eyeball. However, practical application of the trans-pars-planar illumination for true-color retinal imaging is challenging due to the light efficiency delivered through the eyelid and sclera is highly wavelength dependent. In this study, we test the feasibility of true-color retinal imaging using efficiency-balanced visible light illumination and validate multiple spectral imaging (MSI) of the choroid using near infrared (NIR) light illumination.

## Results

### System performance evaluation and pupil walking effect

**Figure 1:**
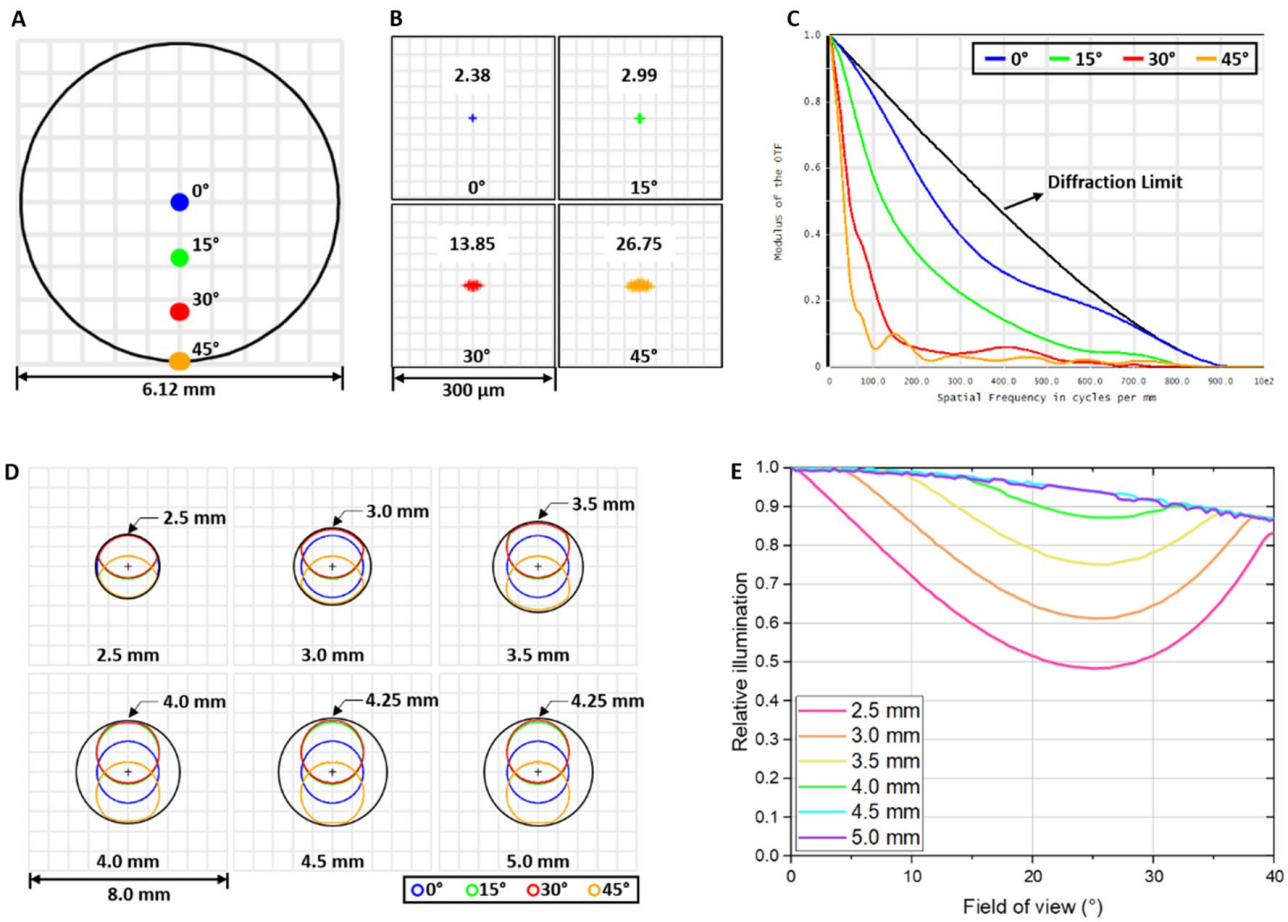
Imaging system performance and pupil walking simulation of proposed fundus imaging system. Simulation of system field of view (**A**), spot diagram (**B**), and modulation transfer function (**C**) at different field angles. Pupil walking depending on eye pupil size. Entrance pupil size and position deformations at the eye pupil plane (**D**) and corresponding relative illumination changes (**E**) from different field angles. Black circles in panel (**D**) show minimum allowable eye pupil size to cover all entrance pupils.

The ray spot locations at the sensor plane were simulated to evaluate the FOV of fundus imaging system (Fig. 1A). The spots from 0° to 45° field angles were positioned in 6.12 mm square at the sensor plane. The maximum field angle can be estimated as ~ 46° considering the camera sensor size is 6.25 mm. Therefore, the maximum FOV is ~ 93° visual-angle (140° eye-angle). The spot diagram simulations of four field angles were shown to characterize the resolution from various field angles (Fig. 1B). The root mean square (RMS) spot radii in each field angle ranged from 2.38 to 26.75 µm. The spot size increased and the shape became ellipse as field angle increase because of ray aberration. The MTF were plotted to characterize the imaging quality (Fig. 1C). The plot showed that ~ 900 cycles/mm can be resolved at all field angles. It can be found that diffraction limited performance was closed at 0° and degraded for nonzero field angles. For general cases, when MTF > 0.3, it is considered to be clearly recognizable, when MTF > 0.6 the image is considered good, and when MTF > 0.8 the image quality is considered very good^24^. The MTFs are 40, 50, 100, and 200 cycles/mm at 0°, 15°, 30°, 45° field angles when MTF > 0.6.

The pupil walking, the entrance pupil position to move or change its size, was observed on eye pupil plane (Fig. 1D). The entrance pupil positions from 15°, 30°, and 45° field angle were shifted to vertical direction. Also, the entrance pupil shapes from 15°, 30°, 45° field angles showed deformation in tangential direction at small eye pupil size and gradually changed to circle shape as eye pupil size increase. The black circles in Fig. 1D showed minimum allowable eye pupil size to cover all entrance pupil from different field angles. This allowable eye pupil reached to maximum at 4.25 mm and did not increase. Note that the maximum entrance pupil size was ~ 2.5 mm at each field angle. The maximum entrance pupil size was determined by the camera aperture size. The camera aperture size was set as 6.66 mm (Camera lens f-number is F/1.8 and focal length is 12 mm). The magnification between lens combination (L1, L2, L3) and triplet lens (L4) was 2.66 based on Zemax simulation. Therefore, the projected camera aperture size on eye pupil plane was ~ 2.5 mm. The relative illumination changes from all field angles were simulated to validate the pupil walking effect (Fig. 1E). The relative illuminations gradually decreased from 100 to 87% as field angle increase at eye pupil size 4.5 mm and 5.0 mm. The relative illuminations were decreased until ~ 25° field angle and reached its minimum then increased at 2.5 mm, 3.0 mm, 3.5 mm, and 4.0 mm eye pupil. Also, the bigger pupil size showed higher relative illumination.

### Spectral efficiency of trans-palpebral illumination

**Figure 2:**
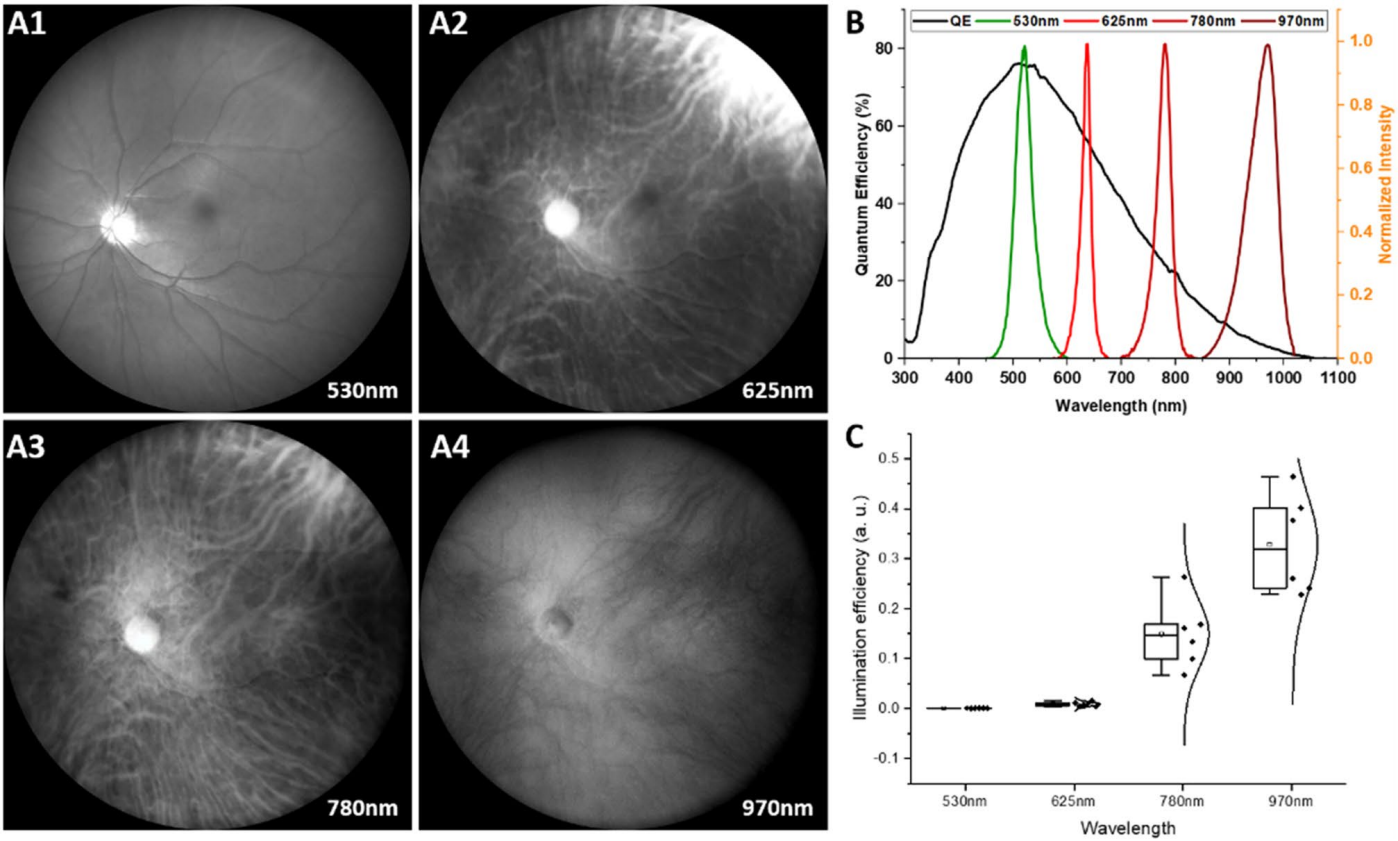
(**A**) Representative wide-field multispectral fundus images from 530 nm (**A1**), 625 nm (**A2**), 780 nm (**A3**), and 970 nm (**A4**) wavelength. (**B**) Light source spectrums and camera quantum efficiency. (**C**) Illumination efficiency from multiple wavelengths.

The representative wide-field fundus images from multiple wavelengths are shown in Fig. 2A. It is observed that the retinal vasculature, including both arteries and veins, were clearly imaged with the 530 nm green light illumination (Fig. 2A1). In contrast, 625 nm red light illumination can visualize the choroidal vasculature (Fig. 2A2). The retinal vasculature was also imaged, but it was not clear as green light illumination. Further enhanced choroidal vasculature was achieved with 780 nm illumination (Fig. 2A3). It showed more choroidal vasculature compared with red light illumination. In 970 nm illumination, choroidal vein structures were selectively imaged (Fig. 2A4).

The spectral efficiency of trans-palpebral illumination was calculated to optimize the illumination of each wavelength. The image brightness *B*_*λ*_ can be estimated at

1 $${B}_{\lambda }\propto {I}_{\lambda }\cdot {P}_{\lambda }\cdot {Q}_{\lambda }{\cdot {G}_{\lambda }\cdot t}_{\lambda }$$

where, *I*_*λ*_ is the illumination efficiency, *P*_*λ*_ is the illumination power, *Q*_*λ*_ is the quantum efficiency of the camera sensor, *G*_*λ*_ is the gain of the camera sensor, and *t*_*λ*_ is the exposure time for each illumination wavelength (*λ*). Therefore, *I*_*λ*_ calculated as,

2 $${I}_{\lambda }\propto {B}_{\lambda }/{P}_{\lambda }\cdot {Q}_{\lambda }\cdot {G}_{\lambda }{\cdot t}_{\lambda }$$

For all illustrated images in this article, the illumination powers were set as *P*_*530*_ = 40 mW, *P*_*625*_ = 18 mW, *P*_*780*_ = 8 mW, and *P*_*970*_ = 4 mW. The quantum efficiencies of the camera sensor are known as *Q*_*530*_ = 75%, *Q*_*625*_ = 58%, *Q*_*780*_ = 22%, and *Q*_*970*_ = 4% (Fig. 2B). The bandwidth of each LED (35 nm, 17 nm, 30 nm, and 60 nm for 530 nm, 625 nm, 780 nm, and 970 nm, respectively) was considered. The illumination powers and quantum efficiencies of single wavelength of each bandwidth were obtained based on Fig. 2B then multiplied and averaged. The gains of the camera sensor were set as *G*_*530*_ = 24, *G*_*625*_ = 10, *G*_*780*_ = 10, and *G*_*970*_ = 10. The exposure times were *t*_*530*_ = 500 ms, *t*_*625*_ = 100 ms, *t*_*780*_ = 100 ms, and *t*_*970*_ = 100 ms. All other camera parameters were maintained as same for all wavelengths. In order to quantify the spectral efficiencies, the averaged pixel value was taken as the *B*_*λ*_ for each illumination wavelength. The illumination efficiencies of each wavelength from seven subjects were plotted in Fig. 2C. The quantitative illumination efficiency was shown in Table 1. The illumination efficiency is highly depending on the wavelength. The higher efficiency at higher wavelength and vice versa. For the easy comparison, the illumination efficiencies of 625 nm, 780 nm, and 970 nm are normalized to that of the 530 nm, i.e., *I*_*λ*_*/I*_*530*_ (Table 2). The normalized illumination efficiencies of 625 nm, 780 nm, and 970 nm are estimated as 30.25, 523.05, 1238.35 times higher than the 530 nm. Student paired t-test was performed between each two group and all p values showed less than 0.01.

**Table 1:** Illumination efficiency of multiple wavelengths.

	530 nm	625 nm	780 nm	970 nm
Subject 1	0.000526	0.015637	0.128292	0.427135
Subject 2	0.000195	0.006887	0.053268	0.270503
Subject 3	0.000148	0.004834	0.124395	0.209732
Subject 4	0.000209	0.013896	0.106439	0.221472
Subject 5	0.000437	0.010597	0.210163	0.369467
Subject 6	0.000284	0.008245	0.13409	0.346982
Subject 7	0.000242	0.003494	0.106528	0.321583
Avg	0.000291**	0.009084**	0.123311**	0.309553**
STD	0.000139	0.004532	0.046829	0.0798

P value: test for difference between each two group. **P < 0.01.

**Table 2:** Illumination efficiency ratio with respect to 530 nm.

	530 nm	625 nm	780 nm	970 nm
Subject 1	1	29.74267	244.0185	812.4318
Subject 2	1	35.30194	273.0453	1386.573
Subject 3	1	32.65722	840.3194	1416.792
Subject 4	1	66.56294	509.8622	1060.889
Subject 5	1	24.2437	480.7901	845.2293
Subject 6	1	29.08339	472.9662	1223.883
Subject 7	1	14.44829	440.4962	1329.759
Avg	1**	33.14859**	465.9283**	1153.651**
STD	0	16.21995	195.5607	251.5291

P value: test for difference between each two group. **P < 0.01.

### Color balance of wide-field fundus image

**Figure 3:**
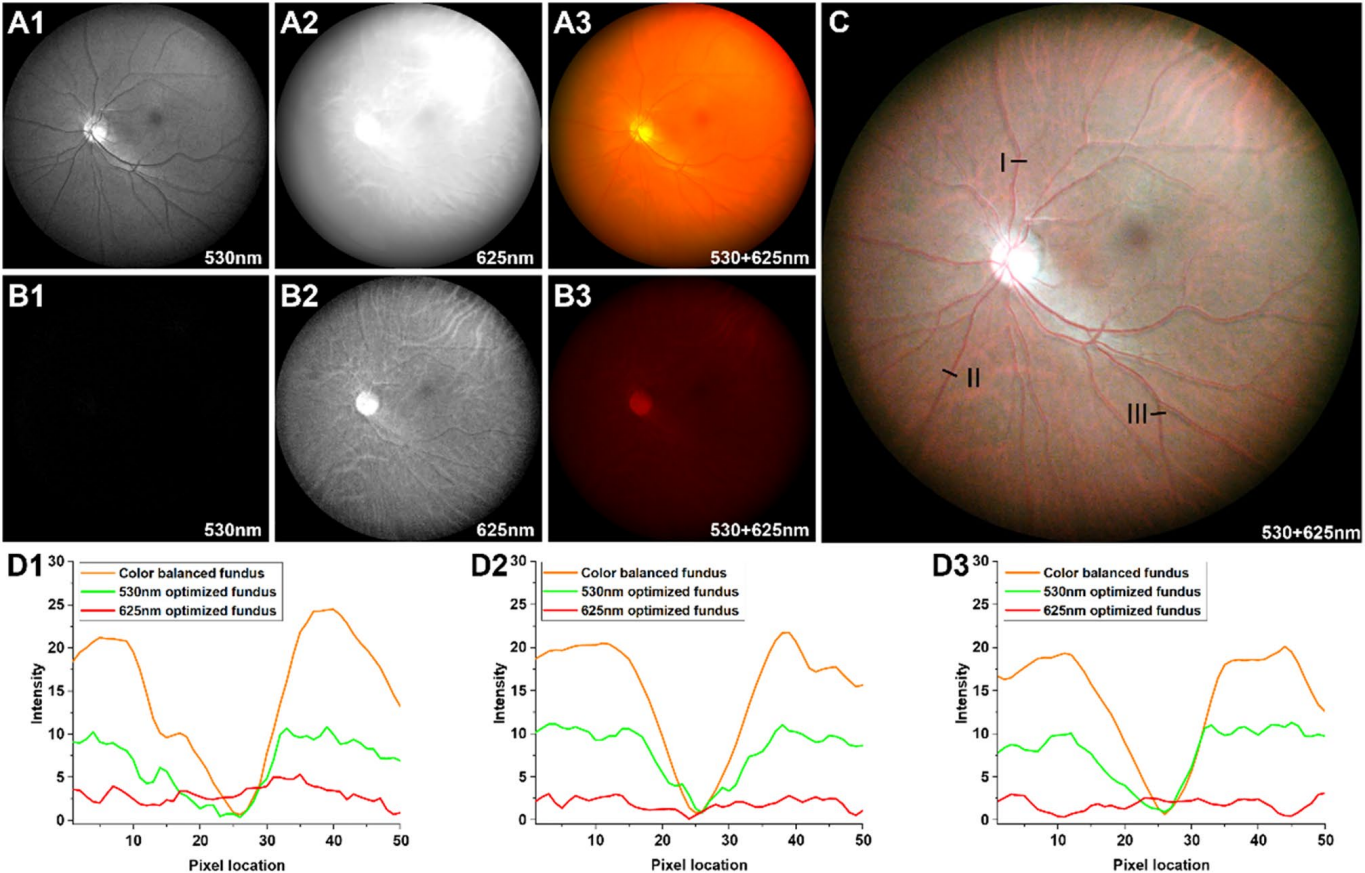
Fundus images with green (**A**) and red (**B**) optimized illumination. Color fundus images (**A3** and **B3**) merged from corresponding green (**A1** and **A2**) and red (**B1** and **B2**) fundus images. Color balanced fundus image (**C**) which is merging of (**A1**) and (**B2**) fundus images. Vessel visibility enhancement of color fundus image from region I (**D1**), II (**D2**), III (**D3**). Each orange, green, and red line indicate the vessel intensity profile from color balanced fundus image (**C**), 530 nm optimized fundus image (**A3**), and 625 nm optimized fundus image (**B3**), respectively.

The color balanced fundus image was constructed to enhance the visibility of the vasculature based on the spectral efficiency (Fig. 3). The green light optimized illumination showed clear retinal vasculature, optic disc and macular region in green fundus image (Fig. 3A1) whereas fundus image was saturated and difficult to see the details in red fundus image (Fig. 3A2). The color fundus image which was merged of Fig. 3A1,A2 showed red dominated fundus image, though optic disc and macular were visualized (Fig. 3A3). The green fundus image did not visualize any noticeable details (Fig. 3B1) and, in the red fundus image, the optic disc, macular and choroidal vasculature was clearly visualized (Fig. 3B2) in red light optimized illumination. The color fundus image of red light optimized illumination showed optic disc and a little choroidal vasculature, however, the image is dim and lose most of detailed structure information (Fig. 3B3). The color balanced fundus image (Fig. 3C) visualized both the retinal and choroidal vasculature clearly compared with non-color balanced fundus images (Fig. 3A3,B3). The quantitative vessel visibility enhancement was evaluated in Fig. 3D. The vessel intensity profile from color balanced fundus image (Fig. 3C) is ~ 2.5 times higher than 530 nm optimized fundus image (Fig. 3A3) whereas no vessel intensity profile was detected in 625 nm optimized fundus image (Fig. 3B3).

**Figure 4:**
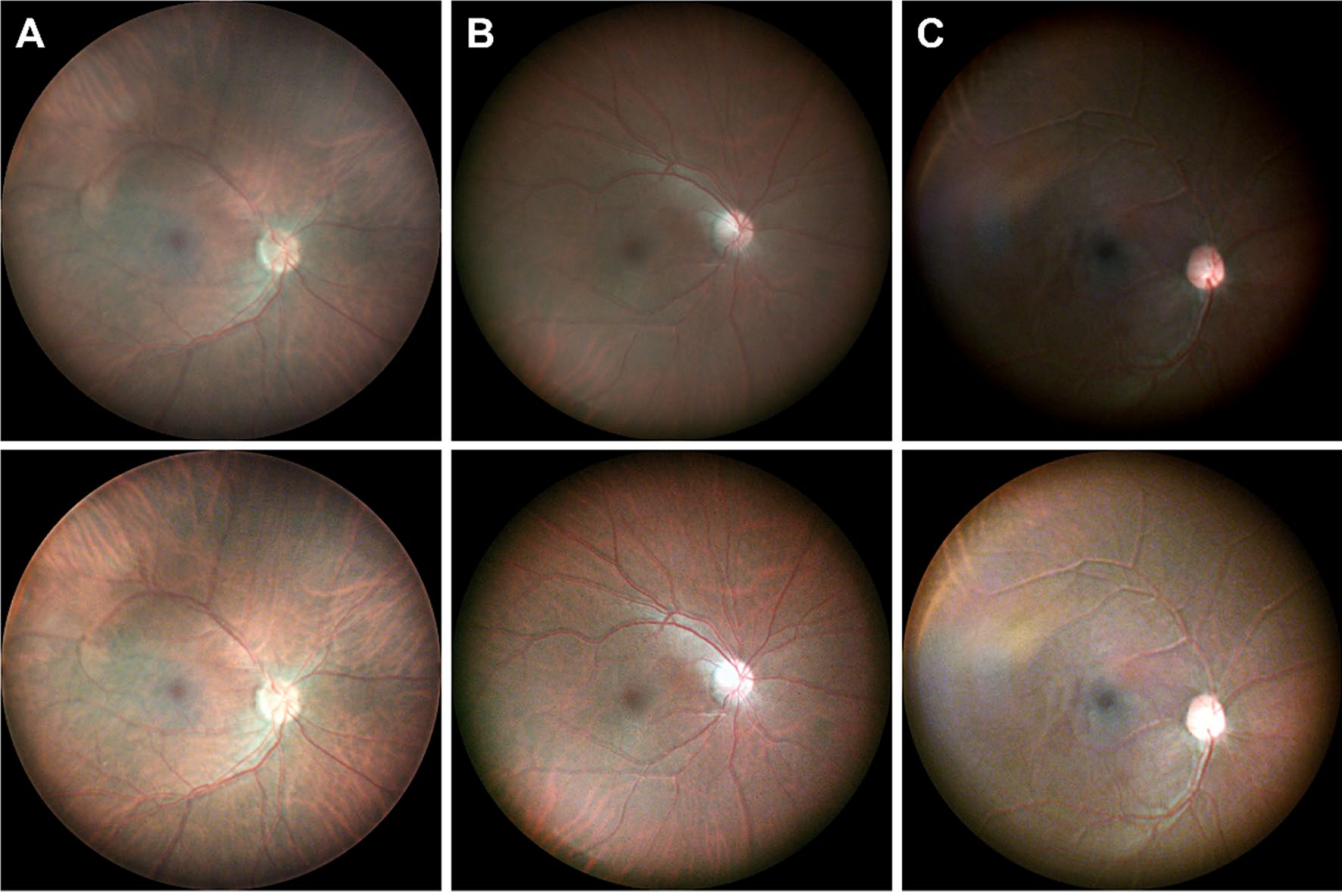
Color balanced fundus images from various pigmentation subjects. Fundus images before (top row) and after (bottom low) normalization from low (**A**), middle (**B**), high pigmentation (**C**) subject.

The fundus images from low, middle, and high pigmentation subjects were acquired to show the pigmentation effect on fundus images (Fig. 4). The pigmentation level affects to overall image brightness (Fig. 4 top row). The macular, optic disc, and retinal vasculature were observed in the fundus images from all pigmentation subjects. The choroidal vasculature gradually become clear from high to low pigmentation subject. Also, clearer retinal and choroidal information was available after the brightness normalization (Fig. 4 bottom row).

### Ultra-wide field fundus image

**Figure 5:**
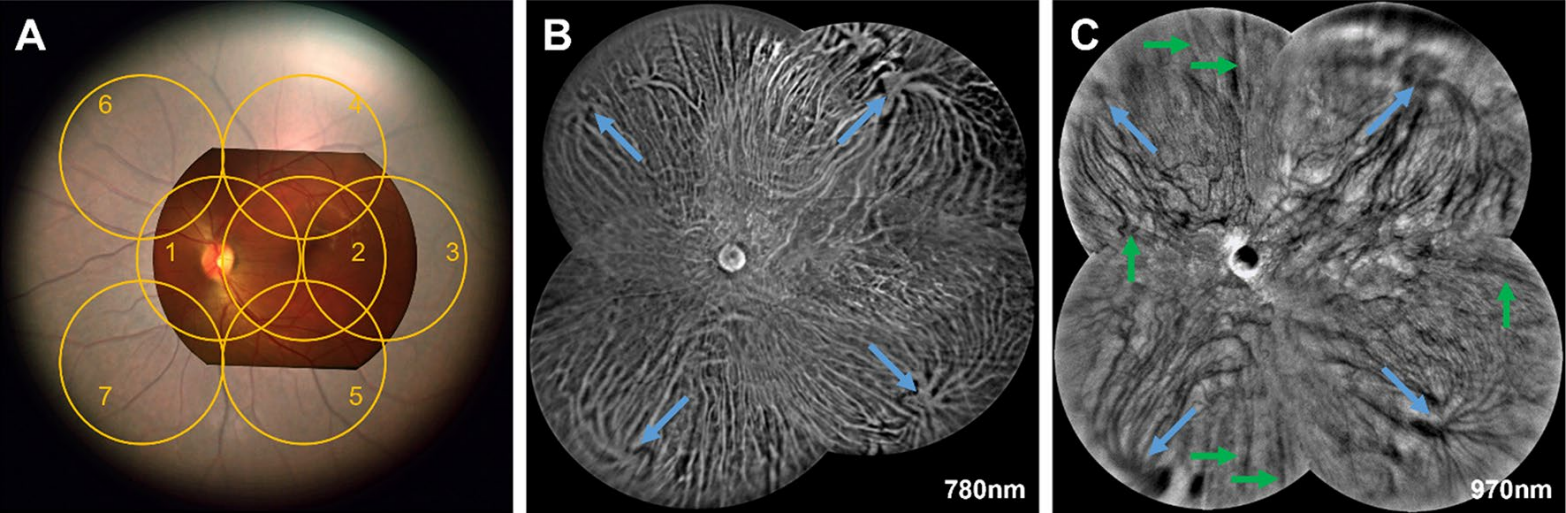
(**A**) Field of view comparison with fundus image from commercial fundus imager Pictor (~ 45° visual-angle; 68° eye-angle). ETDRS seven standard fields (~ 80° visual-angle; 120° eye-angle) covered by fundus image acquired by proposed imaging system. (**B** and **C**) Ultra-wide field choroidal fundus images of 780 nm (**B**) and 970 nm (**C**) by mosaic of five wide field fundus images. The blue and green arrows in (**B** and **C**) show vortex ampullas and ciliary nerves, respectively.

The FOV from the snapshot wide-field fundus image was compared with the fundus image from commercial fundus imager Pictor Plus (VP2RET, Volk Optical Inc., Mentor, OH, USA) in Fig. 5A. The FOV of wide-field fundus image can be measured as ~ 92° visual-angle (138° eye-angle) considering the FOV of fundus image from Pictor Plus is 45° visual-angle (68° eye-angle). The measured FOV of wide-field fundus image is well matched with simulation result in Fig. 1A. The seven standard fields for early treatment diabetic retinopathy study (ETDRS) can be fully covered by the single wide-field fundus image. Also, the fundus image from the prototype fundus camera showed well balanced color compared to commercial fundus camera, and thus has better vessel visibility. The ultra-wide field fundus images were achieved by mosaic of five snapshot wide-field fundus images (Fig. 5B,C). The choroidal vasculature was imaged from the center to the periphery (Fig. 5B). The blue arrows in Fig. 5B indicated to the vortex ampullas which were used as an equator for the 60° visual-angle (90° eye-angle) away from the central retina. Thus, the FOV of the ultra-wide field fundus image can be estimated as > 134° visual-angle (200° eye-angle). In 970 nm NIR fundus image, the choroidal vein structures were visualized in detail (Fig. 5C). The vortex ampullas (blue arrows) were observed and multiple short and long ciliary nerves also observed (green arrows).

## Discussion

**Figure 6:**
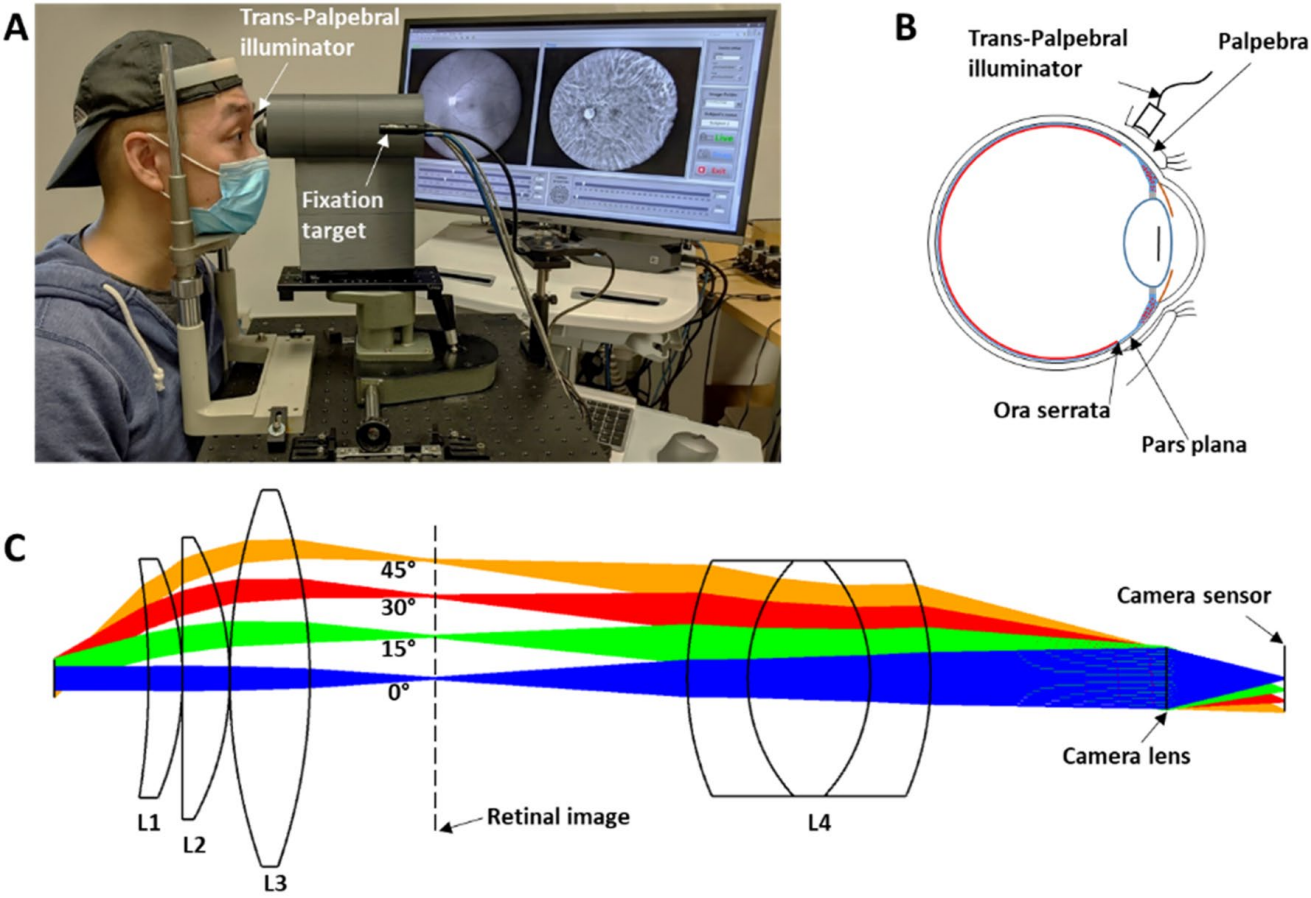
Nonmydriatic wide-field fundus camera with trans-palpebral illuminator for multispectral imaging. (**A**) Photographic illustration of the proposed system. (**B**) Schematic diagram of the trans-palpebral illumination. (**C**) Optical lay out of the proposed system. The field angles in (**C**) represent half of the visual-angle.

The wide-field fundus imaging system was developed for nonmydriatic fundus imaging (Fig. 6A). The trans-palpebral illumination was used to achieve wide-field imaging (Fig. 6A,B). By freeing the entire pupil region for the imaging path, the snapshot FOV was achieved up to ~ 140° eye-angle. Different from the scanning laser ophthalmoscopy (SLO), such as Optos, which involves multiple lasers with different wavelengths and mechanical scanning, this reported fundus camera used LEDs to enable a low-cost, portable device to foster recently emerging telemedicine for rural or underserved areas. We also examined the optical performance of the proposed fundus imaging system through spot diagrams and MTFs (Fig. 1B,C). The RMS spot diagrams which reflect the optical resolution becomes larger as the field angle increase and accompanied by astigmatism (Fig. 1B). As the rays are passing through the different position in the lens with smaller size than the lens diameter (Fig. 6C), the off axis rays strike the lens asymmetrically in tangential and sagittal plane because of lens curvature difference. This produces third-order astigmatism and degrades image quality^25,26^. This degradation was also confirmed by the MTFs (Fig. 1C) which can be used to evaluate imaging quality of the optical imaging system. As the FOV increase, the MTF curve are gradually far from the diffraction limit. The MTF can reflect the most of optical aberrations effects such as spherical aberration, coma, astigmatism, field curvature and distortion^27,28^. We confirmed that most of aberrations are from the distortion, specifically barrel distortion where points in the FOV appear too close to the center, by Seidel coefficients. The distortion considered as geometric misplacement of information and does not reduce the image information^29^. The optical performance of the proposed wide-field fundus imaging system can be improved by adding more lenses for aberration correction. However, this may increase the system complexities, cost and produce other problems. We are currently pursuing a custom lens design and fabrication to improve the optical performance, and thus transit to clinical deployments of the snapshot wide-field fundus image.

The minimum eye pupil size was evaluated as 4.25 mm (Fig. 1D), which can be readily achieved in a dim light condition. The relative illumination showed that the lowest relative illumination is 87% at the 4.0 mm eye pupil size which is normal pupil size in light condition. This result showed that the proposed fundus imaging system can acquire the wide-field image without pharmacological pupillary dilation. The pupil walking effect was observed on the pupil plane. It is induced by spherical aberration of imaging optics and is a common effect in wide angle lenses^30–33^. The lens combination (L1, L2, and L3 lenses) was designed for the wide angle fundus image thus pupil walking was observed. Also, this unique lens combination caused irregular shift of entrance pupil position. The entrance pupil moved to the upper direction at 15° and 30° field angles and to the lower direction at 45° field angle. The entrance pupil shape deformation was also occurred at small eye pupil size and gradually formed a circle shape as eye pupil size increase. This is mainly due to the mechanical vignetting which occurs when light beams emanating from object points located off-axis are partially blocked by external objects such as thick or stacked filters, secondary lenses, and improper lens hoods^34^.

Multispectral fundus images were demonstrated by trans-palpebral illumination (Fig. 2). The green light illumination predominantly shows retinal vasculature while the red and NIR illumination reveals choroidal vasculature. Since the choroid and choriocapillaris sustain the high metabolic rate of the outer retinal layers and retinal pigment epithelium, contributing to the photoreceptor oxygen supply, choroidal imaging can be valuable for clinical management of eye conditions^35,36^. The choroid, located under the retinal pigment epithelium (RPE), is known to have abundant melanin particles to absorb most of the visible light and this absorption is highly depending on the wavelength. Therefore, we investigated the spectral efficiency of trans-palpebral illumination for the imaging optimization and keep the illumination under the safety level. In Table 2, the illumination efficiency ratio showed multiple order higher efficiency in longer wavelength than 530 nm wavelength. This spectral efficiency affected by several factors such as the optical properties of sclera, eyelid, RPE melanin and eye pupil size. Vogel et al.^37^ and Hwang et al.^38^ represented that the light transmittance of human sclera and eyelid is higher at longer wavelength, vice versa. Since the light transmittance of eyelid is affected by pigmentation level to cause image brightness difference (Fig. 4), the subjects with skin type III (light brown) to IV (moderate brown) by Fitzpatrick scale were recruited to minimize the effect of pigmentation level between subjects^39^. Also, RPE melanin absorption decreases as the wavelength increases, and thus the brightness of the image increases. The eye pupil size can be changed depending on the illumination power, which was adjusted differently by each wavelength. The high illumination power reduces the eye pupil, therefore, the brightness becomes dark. Based on the spectral efficiency of trans-palpebral illumination, light efficiency compensated color balanced fundus image was achieved (Fig. 3C). The color characteristics are important to distinguish the features, such as hemorrhage, pigments, or lipids, which may affect the correct diagnosis or staging of eye disease^40,41^. As shown in the Fig. 3D, the color balanced fundus image enhances the vessel visibility. Therefore, accurate color rendering may be vital. Many fundus studies digitally balanced the color ratio by adjusting red and green channel intensities to improve the color characteristics^42^. However, the traditional fundus cameras which use a broad spectrum illumination resulting oversaturated in the red channel and washed out in the green channel thus a retinal image often looks reddish and potentially less informative retinal image. Once it is oversaturated or wash out, it is difficult to recover the information. By the separate controlling of illumination, the dynamic range of red and green channel can be managed individually without affect to each other.

The FOV of snapshot fundus image was compared with fundus image from the commercial fundus imager and ETDRS seven standard fields. (Fig. 5A). A single fundus image covers ETDRS seven standard fields. It is well known that the eye diseases are known to affect both central and peripheral regions of the ocular fundus. Therefore, in this study, we demonstrated the feasibility of ultra-wide field fundus image using trans-palpebral illumination to achieve > 134° visual-angle (200° eye-angle; Fig. 5B,C) FOV. The 780 nm illumination represented the choroid vasculature with vortex ampullas while the 970 nm illumination reveals only large veins with vortex ampullas. The color inversion of veins and background in the 970 nm illumination compared with 780 nm might be the light reflected from the deep sclera, while significant light attenuation occurs at the large vortex veins which exit the globe through the sclera with high flow rate. The vortex vein deformation has been reported in central serous chorioretinopathy and polypoidal choroidal vasculopathy. Thereby it is promising a practical solution to foster objective assessment of choroidal conditions due to eye diseases. Compared with indocyanine green (ICG) which is commonly used in clinics to acquire the choroidal angiography, the reported multispectral fundus imaging is label-free, and thus totally noninvasive without concern about allergic reactions induced by exogenous dye injection. Although, optical coherence tomography (OCT) angiography can visualize the choroidal vasculature, the FOV is relatively smaller than the proposed wide-field fundus images. Also, the ciliary nerves were observed (Fig. 5C). We speculate that the dark edges of the ciliary nerve might result from the light absorption of the ciliary arteries accompanied with the nerve.

## Conclusion

Trans-palpebral illumination enabled a wide-field fundus camera with 93° visual-angle (140° eye-angle) snapshot FOV for MSI. With the aid of a fixation target, ultra-wide field fundus imaging can be readily achieved up to 134° visual-angle (200° eye-angle). Optical performance of the fundus camera was systematically evaluated and the minimum eye pupil size required for nonmydriatic fundus imaging was quantitatively estimated at 4.25 mm. The MSI confirmed that the 530 nm image is predominated by retinal structure, the 625 nm image consists of contributions of both the retina and choroid, the 780 nm image reveals both arteries and veins in the choroid, and the 970 nm image discloses large veins only. In comparison with 530 nm illumination, the 625 nm, 780 nm and 970 nm light efficiencies are 30.25, 523.05, and 1238.35 times higher. The light efficiency compensated 530 nm and 625 nm illumination can be effectively used to enhance image contrast for true-color fundus photography.

## Materials and methods

### Imaging setup

Figure 6A shows photographic illustration of the nonmydriatic wide-field fundus camera with trans-palpebral illumination. The trans-palpebral illuminator consists of 4 optical fibers which has 600 µm diameter and 0.39 numerical aperture (Fig. 6A). Each fiber is connected to LED light sources which has 530 nm (M530L4, Thorlabs Inc, Newton, NJ, USA), 625 nm (M625L4, Thorlabs Inc, Newton, NJ, USA), 780 nm (M780L3, Thorlabs Inc, Newton, NJ, USA), and 970 nm (M970L4, Thorlabs Inc, Newton, NJ, USA). The wavelengths of LEDs were carefully selected to acquire the retinal and choroidal vasculature. The schematic diagram of the trans-palpebral illuminator and eye illustrates the illumination position and pars-plana location (Fig. 6B). The detailed optical layout of the wide-field fundus imaging system is shown in Fig. 6C. The first, second, and third lens (L1, L2, and L3) of the imager are a meniscus lens (LE1234-A, Thorlabs Inc., Newton, NJ), plano convex lens (67–152, Edmund Optics Inc., Barrington, NJ), and double convex lens (63–688, Edmund Optics Inc., Barrington, NJ, USA), respectively. This lens combination produces an aerial image of the retina in front of the relay optics, triplet achromatic lens L4 (67–422, Edmund Optics Inc., Barrington, NJ, USA). In coordination with the relay optics and a camera lens with a focal length of 12 mm (33–303, Edmund Optics Inc., Barrington, NJ, USA), the aerial image is relayed to the camera sensor. A color CCD camera (GS3-U3-41S4C-C, Flir systems Inc, Wilsonville, OR, USA) and a monochrome camera (GS3-U3-41S4M-C, Flir systems Inc, Wilsonville, OR, USA) were used for MSI (Figs. 2, and 5) and color fundus imaging (Fig. 3), respectively. Both cameras have a frame rate of 18 frames per second, frame resolution of 2016 × 2016 pixels, with 3.1 μm × 3.1 μm pixel size. The sensor provides quantum efficiencies of 75%, 58%, 22%, and 4% at 530 nm, 625 nm, 780 nm, and 970 nm wavelength, respectively.

### Optical simulation

The optical system for the wide-field fundus imaging (~ 93° visual-angle; ~ 140° eye-angle) was designed and evaluated by Zemax simulation (Zemax OpticStudio 18.7, ZEMAX LLC., Kirkland, WA, USA) to optimize the image quality and to ensure optimum performance. As shown in Fig. 6C, the simulation starts from the eye pupil. All off-the-shelf lenses were selected from the lens catalog in Zemax libraries (Fig. 6C). Paraxial surface was used for last lens surface to mimic the camera lens which is not available in the lens catalog and also the aperture was set to working as a stop to simulate ray vignettes. The system optimization was performed by field of view, spot diagram, and modulation transfer function (MTF) whose modulus of optical transfer function evaluation from various field angles at 0°, 15°, 30°, and 45° visual-angle (0°, 23°, 45°, and 68° eye-angle). Note that the field angle is half of the visual-angle. The minimum eye pupil size for the wide-field fundus image was validated by simulating the entrance pupil shape and position deformation from various field angles. The eye pupil size was changed from 2.5 to 5 mm diameter with fixed camera aperture size as 6.66 mm. To validate the effect of eye pupil size on the image quality, the relative illumination was simulated.

### Human subjects and fundus imaging

This study was approved by the Institutional Review Board of the University of Illinois at Chicago and followed the ethical standards stated in the Declaration of Helsinki. Seven healthy subjects with no history of eye disease were recruited to validate the proposed fundus camera prototype. The informed consent was taken from each subject. The informed consent for publication of identifying image (Fig. 6A) in the online open-access publication was obtained from the subject.

The fundus images were taken in a dark room condition. The subject head was placed to forehead/chin rest for stable imaging (Fig. 6A). The trans-palpebral illuminator was positioned to the eyelid (Fig. 6A,B). The illuminator can adjust transverse, vertical position, and angle depending on the subject. Considering the pars-plana width and distance from the limbus, the center of illuminator was placed ~ 6 mm away from the limbus and the orientation of the illumination was aligned to normal direction of the sclera. The optimal illumination location, i.e., the pars-plana, could be identified based on the image quality by fine adjusting of the illuminator. During the image acquisition, live view of fundus image was streamed to monitor imaging location and performed the fine focus adjustment. The illumination location was maintained during the multispectral fundus imaging. The exposure times were set as 500 ms and 100 ms for 530 nm and rest of other wavelength LEDs, respectively. For the color fundus image, 530 nm and 625 nm LED was turned on and off sequentially for green and red fundus image, respectively. The camera exposure was set as same for all imaging sequence except for the high pigmentation subject (Fig. 4A). The exposure time was increased twice for high pigmentation subject than middle and low pigmentation subjects. And the illumination power was adjusted to 40 mW and 1.5 mW for green and red optimized fundus images. For the five fundus images were acquired from different location to expand the FOV of MSI by utilizing fixation target. First, center aimed fundus was acquired and other four image locations were positioned roughly 33°–40° visual-angle (50°–60° eye-angle) away from the center fundus image to each diagonal direction.

### Ocular light safety

The ocular light safety was evaluated according to ISO standard “Ophthalmic Instruments—Fundus Cameras” (10940:2009)^43^ which safety limits are at least 10 times lower for retinal threshold damage. Both photochemical and thermal hazards of the retina were quantitatively evaluated. To acquire the fundus images within the safety limit, maximum permeable exposure time was calculated from all wavelengths. The illumination power of each wavelength was 40 mW, 15 mW, 10 mW, and 4 mW for 530 nm, 625 nm, 780 nm, and 970 nm, respectively. The transmission of the sclera and eyelid for different wavelengths were considered. According to the ISO standard, a maximum of 10 J/cm^2^ weighted irradiance is allowed on the retina without photochemical hazard concern. The weighted irradiance was calculated using the photochemical hazard weighting function provided in the ISO standard. For conservative estimation of the worst case, assuming all light directly reaches to the retina behind the illuminated sclera area, the illuminated retinal area was estimated as 0.2826 mm^2^ considering that the fiber diameter is 600 µm. The details about the retina safety calculation were described in ref.^14^. The maximum permeable exposure time was ~ 35 min for the 530 nm illumination and > 24 h for the rest of wavelengths. The maximum weighted power intensity allowed on the sclera without thermal hazard concern is 700 mW/cm^2^. The equivalent powers for thermal hazard estimation were 191 mW/cm^2^, 127 mW/cm^2^, 154 mW/cm^2^ and 62 mW/cm^2^ for 530 nm, 625 nm, 780 nm and 970 nm light sources, respectively, which is 4–11 times below compared with the maximum weighted power intensity allowed on the sclera without thermal hazard concern.

### Disclosures

D. Toslak and X. Yao have patent applications relative to wide-field fundus photography.

## Data Availability

Data underlying the results are presented in this article. Additional information may be obtained from the corresponding author upon reasonable request.
